# mTORC1 and mTORC2 are differentially engaged in the development of laser-induced CNV

**DOI:** 10.1186/s12964-019-0380-0

**Published:** 2019-06-14

**Authors:** Jin Young Yang, Sanjar Batirovich Madrakhimov, Dong Hyuck Ahn, Hun Soo Chang, Sang Joon Jung, Seung Kwan Nah, Ha Yan Park, Tae Kwann Park

**Affiliations:** 1 Department of Interdisciplinary Program in Biomedical Science, Soonchunhyang Graduate School, Bucheon Hospital, Bucheon, South Korea; 2 Laboratory for Translational Research on Retinal and Macular Degeneration, Soonchunhyang University Hospital Bucheon, Bucheon, South Korea; 3 Department of Medical Bioscience, Graduated School, Soonchunhyang University, Bucheon, South Korea; 4 Department of Ophthalmology, Soonchunhyang University Hospital Bucheon, #170, Jomaru-ro, Wonmi-gu, Bucheon, 14584 South Korea; 5 Department of Ophthalmology, College of Medicine, Soonchunhyang University, Cheonan, Choongchungnam-do South Korea; 6 Department of Ophthalmology, College of Medicine, Soonchunhyang University, Bucheon Hospital, Bucheon, South Korea

**Keywords:** Age-related macular degeneration, Choroidal neovascularization, Sirolimus (rapamycin), mTORC1, mTORC2

## Abstract

**Background:**

The mechanistic target of rapamycin (mTOR) pathway is a potential target to inhibit pathologic processes in choroidal neovascularization. However, the exact role of mTOR signaling in the development of CNV remains obscure. In this study, we assessed the role of mTORC1 and mTORC2 as well as the effect of rapamycin (sirolimus) on choroidal neovascularization (CNV) in a laser-induced mouse model.

**Methods:**

In experiment A, we observed the natural course of CNV development and the dynamics of mTOR-related proteins during the 12 days after the laser injury. The expression of mTOR-related proteins was evaluated using Western blot (WB). Cryosections of CNV-induced mice were immunostained for the visualization of the vascular and extravascular components of the CNV. Experiment B was performed to confirm the critical period of mTOR signaling in the development of laser-induced CNV, we administered rapamycin before and/or during the active period of mTOR complexes. WB and immunofluorescence staining was performed to evaluate the mode of action and the effect of mTOR inhibition on CNV development.

**Results:**

In experiment A, we detected high levels of p-mTOR S2448 and p-mTOR S2481 from the 5th to 12th day of laser injury. Immunofluorescence imaging of cryosections of mice sacrificed on day 7 revealed greater co-immunoreactivity of p-mTOR S2448 positive cells with CD11b and F4/80, while p-mTOR S2481 positive cells showed colocalization with CD31, α-SMA, and cytokeratin. In experiment B, rapamycin injection during the active period of mTOR signaling demonstrated near-complete inhibition of CNV lesion as well as significant induction of autophagy.

**Conclusion:**

Our study suggests the mTOR as a critical player during CNV development in laser-induced mouse model through differentially acting with the mTORC1 and mTORC2. mTORC1 activity was high predominantly in inflammatory cells in CNV lesion, while mTORC2 activity was higher in vascular components and the RPE.

**Electronic supplementary material:**

The online version of this article (10.1186/s12964-019-0380-0) contains supplementary material, which is available to authorized users.

## Background

Age-related macular degeneration (AMD) is an acquired multifactorial disease among the elderly population. As being responsible for 10% of the blindness of people aged 65 and older, AMD has a leading position among the causes of irreversible blindness [1, 2].

AMD clinically manifests in 2 forms: non-exudative (non-neovascular, “dry”) and exudative (neovascular, “wet”). Wet-AMD has the worse prognostic outcome in terms of vision [1, 3, 4]. The precise pathophysiological mechanisms of wet-AMD remain unknown. It is generally accepted that under the influence of metabolic, functional, genetic and environmental factors, lipofuscin containing cellular inclusions accumulate in retinal pigment epithelium (RPE), leading to the dysfunction of RPE cells and Bruch’s membrane. ‘Excessive damage of Bruch’s membrane and upregulation of proangiogenic factors result in sprouting of abnormal choroidal vessels – choroidal neovascularization (CNV). Abnormal vessels cause exudation, hemorrhage, fibrosis and outer retinal degeneration [5–7].

Various regulatory mechanisms are involved in the development of CNV. The vascular endothelial growth factor (VEGF) is the most investigated among other factors contribute to CNV development and currently, VEGF targeted therapy is a primary treatment option for CNV [8–11]. However, a number of patients may demonstrate a worsening course of the disease even with an aggressive approach [11, 12], suggesting other regulatory mechanisms contribute to CNV formation. The search for alternative pathways revealed a potential factor – mTOR – in the regulation of pathogenesis of wet-AMD [13].

mTOR is the target of antifungal antibiotic – rapamycin – which is macrolide known for antiproliferative properties. mTOR is an atypical serine/threonine protein kinase and part of the phosphoinositide 3-kinase (PI3K)-related kinase family. mTOR functions in two different protein compounds – mTOR complex 1 and 2 (mTORC1 and mTORC2) [14]. Several in vivo studies have demonstrated the therapeutic effect of the mTOR pathway inhibition in retinal neovascular diseases, including wet-AMD, proliferative diabetic retinopathy and retinopathy of prematurity [15–19]. It has been published, that process of pathological angiogenesis includes activation of mTOR pathway selectively in proliferative state endothelial cells (ECs) and mTOR inhibitors target these cells [15], suggesting that mTOR is a potential target for the treatment of wet-AMD. Although, most studies reported the therapeutic effect of mTOR inhibition, the exact function of mTORC1 and mTORC2 in ocular pathological angiogenesis remains unknown. In contrast to previous studies, where the focus of the research was the inhibition of mTORC1, we recently demonstrated that dual inhibition of mTORC1 and mTORC2 via rAAV-mTOR short hairpin RNA leads to significant regression of CNV in a laser-induced mouse model [20]. In the current study, we evaluated the role of the mTOR pathway in the natural course of laser-induced CNV in mice. During the subacute stage of CNV development, mTORC1 was active in inflammatory cells in CNV lesion, while mTORC2 activity was predominantly observed in ECs, pericytes and the RPE. These results suggest that the mTOR pathway contributes to CNV development differentially effecting on inflammation and angiogenesis through mTORC1 and mTORC2. To evaluate the mode of action and the effect of mTOR inhibition on CNV development, we used rapamycin, which inhibits the mTOR pathway immediately after administration and equally effects on all cell types, unlike gene-based drugs.

## Materials and methods

### Animal care

All animal experiment was designed and conducted in accordance with the Guide of the Care and Use of Laboratory Animals, the Association for Research in Vision and Ophthalmology Statement for the Use of Animals in Ophthalmic and Vision Research, and approved by the Institutional Animal Care and Use Committee for Soonchunhyang University Hospital Bucheon.

Mice used in this study were C57/BL6 strain (8 weeks, male, 22~24 g) and purchased from the Orient Bio Inc., (Seongnam, South Korea). All mice were housed in breeding cages in a room with a 12/12-h light/dark cycle and had free access to food and water. The humidity and temperature were respectively maintained at 50% and 23~26 °C.

### Laser-induced CNV in mice

For the induction of CNV, mice were anesthetized by intraperitoneal injection using the mixture of Zoletil 50 (Virbac, Carros Cedex, France) and Rompun (Bayer Healthcare, Leverkusen, Germany). A mixture of 0.5% tropicamide and 0.5% phenylephrine (Tropherine) (Hanmi Pharm, Seoul, South Korea) was instilled for pupil dilation. Laser photocoagulation (spot diameter-200 μm, duration-20 ms, power-120 mW, 5 spots/eye) was performed using a PASCAL diode ophthalmic laser system (neodymium-doped yttrium aluminium garnet [Nd:YAG], 532 nm; Topcon Medical Laser Systems, Livermore, CA) and laser burns are produced in the 2, 4, 7, 10 and 12 o’ clock position around the optic disc, with the laser focused on the RPE. The presence of a bubble confirmed the disruption of Bruch’s membrane.

### Experimental design and samples harvesting

In experiment A, 50 mice were assigned to 5 groups: 1) Normal group, 2) CNV3d, 3) CNV5d, 4) CNV7d and 5) CNV12d regarding the day of sacrifice after CNV induction.

Experiment B included 60 mice, divided into 6 groups: 1) Normal, 2) 5dRapa (−), 3) 5dRapa(+), 4) 12dRapa(−), 5) 12dRapa(+), 6) 5dRapa(−)/7dRapa group. Except for the normal group, all mice induced CNV. To compare the preventive and therapeutic effect of rapamycin on laser-induced CNV, the rapamycin injected mice were divided into 5dRapa(+), 12dRapa(+), and 5dRapa(−)/7dRapa(+). The mice in the 5dRapa(+) and 12dRapa(+) group received daily intraperitoneal injections of rapamycin (Sigma-Aldrich, St. Louis, MO) diluted in 4% ethanol and 5% Tween-20 in distilled water (3 mg/ml) for 5 days and 12 days, respectively. In the 5dRapa(−)/7dRapa(+) group, rapamycin was administered for 7 days after day 5 of laser photocoagulation. Animals were sacrificed after photocoagulation on days 5 and 12 according to the experimental group.

After completing the animal experiment, six mice (12 eyes) from each group were used for WB and four mice (8 eyes) for histologic analysis. For histologic analysis, mice were deeply anesthetized and perfused intracardially with 0.1 M phosphate buffer (PB) containing 1000 U/ml of heparin, followed by an infusion of 4% paraformaldehyde (PFA) in 0.1 M PB. The eyecup was made by removing the anterior segment from the enucleated mouse eye and fixed with 4% paraformaldehyde (Biosesang, Seongnam, South Korea) for 1 h, dehydrated in 30% sucrose overnight, and embedded in frozen section compound (Leica Biosystems Richmond, IL). The sample collection for the WB experiment was that the neuroretina and RPE/choroid (RPE/Ch) layer was separated from the enucleated mouse eyes and stored at − 80 °C until the experiment.

### Fundus fluorescent angiography (FFA)

In all animal experiments, FFA images were taken before sacrifice. Before the Fundus Fluorescent Angiography (FFA), mice were anesthetized and pupils were dilated. FFA images were taken at appropriate intervals for 5 min after intraperitoneal injection of 2% fluorescein sodium (Fluorescite; Akorn, Lake Forest, IL), using a scanning laser ophthalmoscope (Heidelberg Retina Angiograph 2; Heidelberg Engineering, Heidelberg, Germany).

The FFA images taken at similar time points were analyzed using ImageJ software (National Institutes of Health, Bethesda, MD) by manual selection of the maximal border of the leakage area. Areas and intensity were calculated in pixels and the area values were converted to μm^2^ using the “Scale” tool.

### Western blot

We studied the levels of proteins separately in neuroretina and RPE/Ch. Protein levels of neuroretina or RPE/Ch from one eye is not sufficient to detect all indicated antibodies. Therefore, we pooled the neuroretina from both eyes; the same was done for RPE/Ch. Tissue was disrupted in RIPA II lysis buffer (Gendepot, Barker, Tx) containing Xpert phosphatase inhibitor cocktail (Gendepot, Barker, Tx) and Xpert protease inhibitor cocktail (Gendepot, Barker, Tx) for 1 h on the ice. After centrifuge (13,000 rpm, 15 min, 4 °C), insoluble material was removed and the only supernatant was obtained. Protein lysate was quantified using Pierce BCA Protein Assay kit (Thermo scientific, Middlesex, MA) according to the manufacturer’s instruction.

The lysates were solubilized in Laemmli sample buffer (4X) (Gendepot, Barker, Tx), boiled for 10 min at 95 °C. Aliquots of each sample with an equal amount of protein (10 μg) were separated using SDS-acrylamide gel in the range from 6 to 12%, depending on the molecular weight of the target antibody and transferred onto PVDF membrane (ATTO, Amherst, NY). The membranes were blocked with 5% skim milk in PBS containing 0.1% Tween-20 for detection of total proteins and 5% BSA in PBS containing 0.1% Tween-20 for phosphorylated proteins for 1 h at room temperature and incubated with anti-mTOR, anti-VEGFR2, anti-phospho-S6 ribosomal protein (S240/244), anti-AKT, anti-phospho-AKT (S473), anti-ERK, anti-phospho-ERK from Cell signaling technology (Danvers, MA), anti-phospho-mTOR (S2448), anti-phospho-mTOR(S2481), anti-VEGFA, anti-VEGFR1 from Abcam (Cambridge, UK), anti-HIF1α, anti-β-actin, from Santa Cruz Biotechnology (Dallas, TX), anti-LC3B (Novus Biologicals, Littleton, CO) antibodies overnight at 4 °C; details on antibodies are given in Additional file 2: Table S1. After washing the membranes, they were incubated with horseradish peroxidase (HRP)-conjugated goat anti-rabbit IgG and goat anti-mouse IgG (Genedepot, Barker, Tx) at room temperature for 2 h. The immunoreactive signal was developed using Western blotting detection kit (WesternBright ECL) (Advansta, San Jose, CA). Bands on blots were quantified using the ImageJ software (National Institutes of Health, Bethesda, MD).

To quantify LC3II/LC3I ratio, we analyzed the rows of LC3I and LC3II separately using ImageJ and normalized the values against β-actin values. Then, the values for LC3II of each lane over LC3I values were measured.

### Immunofluorescence

Fresh eyecup cryosections (10 μm in thickness) were made with a Cryotome (Thermo Fisher Scientific Shandon Cryotome, Middlesex, MA). The slides were washed with 1XPBS containing 0.1% Triton X-100 (Sigma-Aldrich, St. Louis, MO) and blocked with 5% donkey serum in PBST for 1 h. The blocked slides were then incubated with anti-phospho-mTOR (S2448), anti-phospho-mTOR (S2481) from Abcam (Cambridge, UK), anti-α-SMA (Sigma-Aldrich, St. Louis, MO), anti-CD31 (Invitrogen Corp, Carlsbad, CA), anti-cytokeratin (Dako, Santa Clara, CA), anti-F4/80, anti-CD11b from Bio-rad (Hercules, CA), anti-ATG9A (Novus Biologicals, Littleton, CO) antibodies for 2 h and detected with secondary antibodies (Alexa Fluor 488 or 568, dilution 1:1000; Invitrogen Corp., Carlsbad, CA), including Hoechst 33342 (Invitrogen Corp, Carlsbad, CA). After washing, the sections were mounted with fluorescence mounting medium (Dako, Santa Clara, CA). All slides were imaged using a Zeiss ZEN LSM 710 confocal microscope (Carl Zeiss, Oberkochen, Germany) at × 20 and × 40 magnification.

For RPE/Ch whole mounts, neuroretina was removed from neuroretina/RPE/Choroid complex and was submerged in 70% ethanol for 30 min. Then tissue was washed using 0.1% 1XPBST for 30 min, blocked using 5% goat serum in 1XPBS for 3 h, treated with p-mTOR S2448 and p-mTOR S2481 (Abcam, Cambridge, UK) antibodies at 4 degree overnight. Next day, the tissues were exposed to secondary antibody for 4 h and nuclear counterstaining with Hoechst 33342 (Invitrogen Corp, Carlsbad, CA) at room temperature. The whole mounts were mounted with fluorescence mounting medium. All slides were imaged using a Zeiss ZEN LSM 710 confocal at × 20 and × 40 magnification.

### Statistical analysis

In experiment A, FAG results were analyzed by a hierarchical mixed ANOVA model considering the time points as a fixed effect, mouse and eyes variability as random effects [21]. Post hoc analysis was performed using Dunnet’s post hoc test. In experiment B, the same mixed model was used considering the treatment regimen and time points as a fixed effect, mice and eyes variability as random effects. Statistical calculations was done using SPSS software (version 20.0 for Windows; IBM Corp., Armonk, NY).

Statistical significance was calculated with Kruskal-Wallis H test for comparisons of groups in WB experiments and followed by post hoc analysis (Mann-Whitney U test) using SPSS software. Data are presented as the mean ± S.E.M.

## Results

### mTOR pathway in the natural course of laser-induced CNV model in mice

**Fig. 1:**
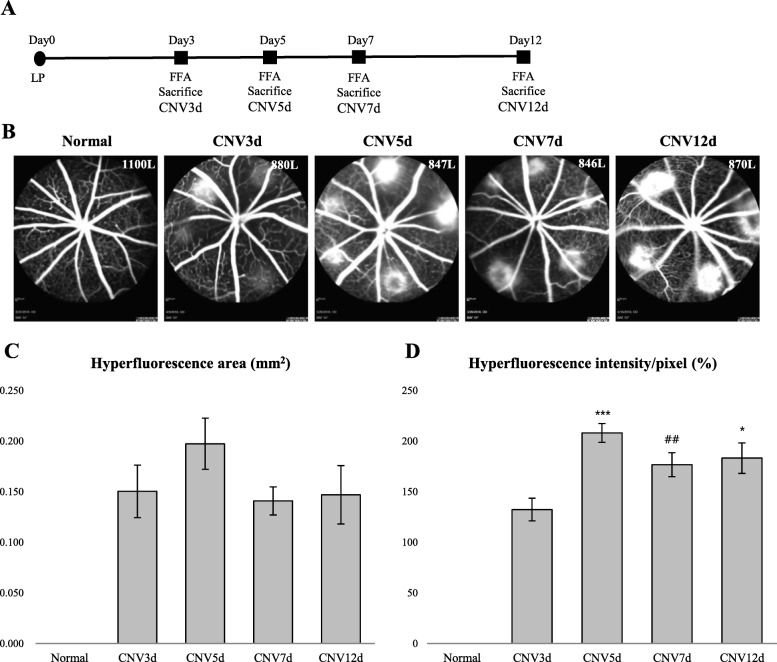
Experiment **a** design and Fluorescein angiographic analysis of laser-induced CNV. **a** – A schematic diagram for experimental procedures. **b** – Representative FFA images of CNV lesions captured at day 3, 5, 7 and 12 after laser induction. **c**, **d** – quantitative analysis of FFA images for hyperfluorescence area and intensity. Data are presented as the mean hyperfluorescence area/pixel ± S.E.M. and the mean hyperfluorescence intensity/pixel (%) ± S.E.M. * *P* ≤ 0.05, ** *P* ≤ 0.005, ***P ≤ 0.001, Mann-Whitney U test, compared with CNV3D group. # *P* ≤ 0.05, ## *P* ≤ 0.005, ###*P* ≤ 0.001, Mann-Whitney U test, compared with CNV5D group

In experiment A, we observed natural time-course of CNV formation for 12 days after the laser treatment. FFA, performed on the 3rd day after LP, revealed some hyperfluorescent lesions (Fig. 1b). Well-demarcated hyperfluorescent spots were observed in all laser photocoagulation zones on day 5, indicating the establishment of CNV lesion (Fig. 1b). Analysis of FFA images demonstrated a decrease in hyperfluorescence area (*P* = 0,169) and intensity value (*P* ≤ 0.05) at the day 7 of the follow-up comparing to the 5th-day results (Fig. 1b-d).

**Fig. 2:**
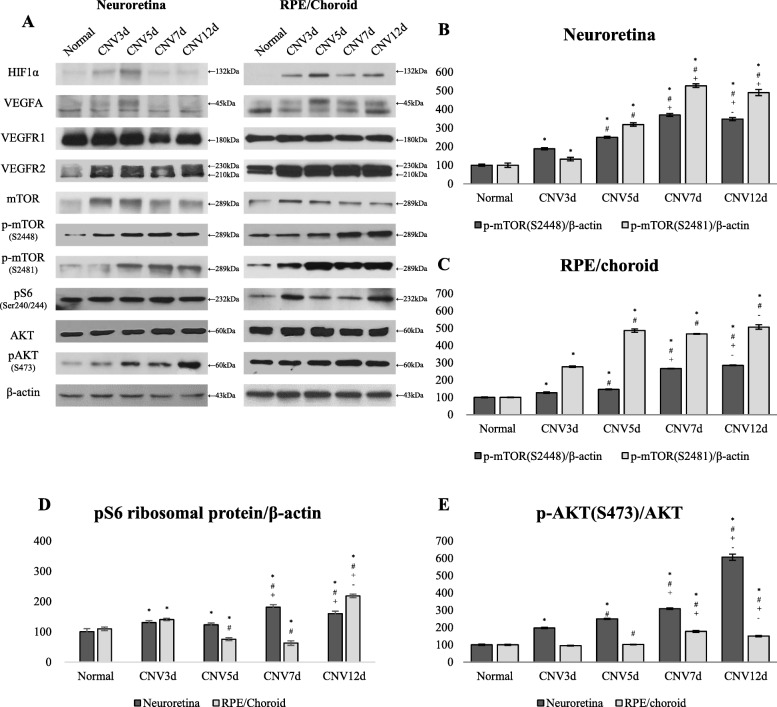
The expression of HIF1α, VEGFA, VEGFR1/2, and mTOR-related proteins detected by Western blot. **a** – Expression of indicated proteins. **b**, **c** – comparison of expression of p-mTOR S2448 and S2481 relative to total mTOR levels in NR and RPE/Choroid. **d**, **e** – relative expression of the pS6 ribosomal protein and pAKT (S473) normalized to β-actin. Data are presented as the mean ± S.E.M. * *P* ≤ 0.05, Mann-Whitney U test, compared with Normal group. # *P* ≤ 0.05, Mann Whitney U test, compared with CNV3d group. + *P* ≤ 0.05, Mann Whitney U test, compared with CNV5d group. - *P* ≤ 0.05, Mann Whitney U test, compared with CNV7d group

Expression of HIF1α and VEGF-related proteins in laser-induced CNV was evaluated using WB. HIF1α levels were upregulated and peaked on day 5 after laser treatment in both neuroretina (NR) and RPE/Choroid (RPE/Ch) (Fig. 2a). Corresponding to HIF1α levels, VEGFA demonstrated the same activation pattern (Fig. 2a, upper lane). VEGFR2 levels were increased by laser treatment at day 3 and kept high up to day 12 (Fig. 2a). WB for VEGFR1 did not show significant changes in expression levels during the whole observation period in either NR or RPE/Ch.

To evaluate mTORC1 and mTORC2 activity, we analyzed the levels of mTOR phosphorylation on S2448 and S2481 in NR and RPE/Ch of mice at indicated time points after laser treatment using WB. At day 3, p-mTOR S2448 expression was significantly upregulated, however, higher values were observed in both NR and RPE/Ch from 5th to 12th day, demonstrating a similar trend to that of p-mTOR S2481 (*P* ≤ 0.05, Fig. 2a-c).

Next, we estimated the expression of pS6 - a downstream target of mTORC1, which was gradually induced and the highest expression was observed on day 7 and 12 in NR. However, analysis of RPE/Ch showed two significant peaks: on day 3 and 12 (Fig. 2a, d). Laser treatment upregulated the expression of the downstream target of mTORC2 - p-AKT S473 and the values from the 5th to 12th day were significantly high in both NR and RPE/Ch (Fig. 2e).

**Fig. 3:**
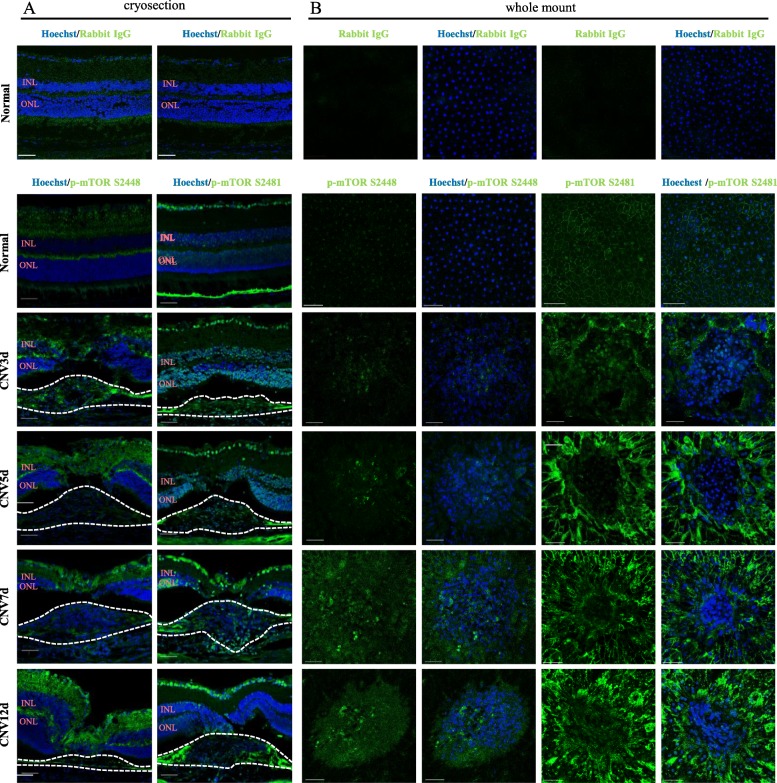
Cryosections (**a**) and whole mounts (**b**) of the mouse retina with laser-induced CNV lesions were immunostained for p-mTOR S2448 and S2481. Scale bar =50 μm

Immunolocalization of phosphorylated mTOR at S2448 and S2481 in laser-induced CNV was examined in neuroretina/RPE/choroid/sclera complexes isolated from mice after 3, 5, 7 and 12 days of laser injury and in control. Following the laser injury, p-mTOR S2448 label gradually increased in the CNV lesion and increased beyond the lesion without changes in the distribution pattern. The strong p-mTOR S2481 signals appeared in outer and inner nuclear layers, also signals substantially increased in the RPE, choroid, and ganglion cell layers (Fig. 3a). In addition, confocal images of the whole mounts demonstrated the gradually increased expressions of p-mTOR S2448 inside the CNV lesion at the level of RPE layer. Meanwhile, p-mTOR S2481 expression in the whole mounts was intense from day 5 to day 12. (Fig. 3b).

**Fig. 4:**
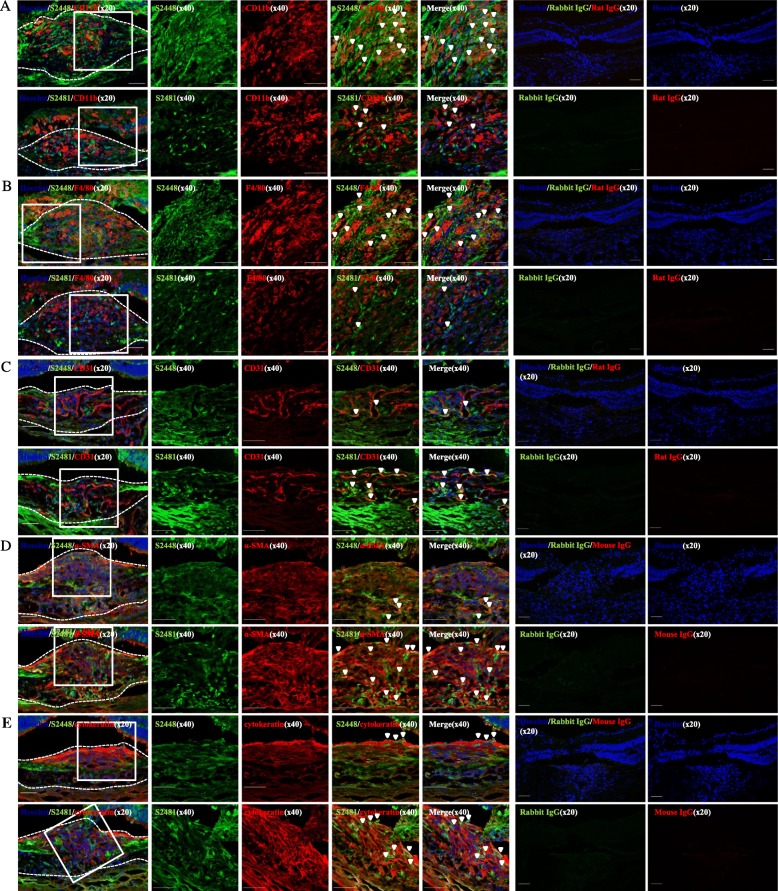
Representative confocal images of cryosections obtained on day 7 after laser photocoagulation co-stained for inflammation−/angiogenesis-related markers with p-mTOR S2448 or S2481. **a** – Immunofluorescence staining for CD11b with p-mTOR complexes. Scar b ar = 50 μm. **b** - Immunofluorescence staining for F4/80 with p-mTOR complexes. Scar bar = 50 μm. **c** - Immunofluorescence staining for CD31 with p-mTOR complexes. Scar bar = 50 μm. **d** - Immunofluorescence staining for α-SMA with p-mTOR complexes. Scar bar = 50 μm. **e** - Immunofluorescence staining for cytokeratin with p-mTOR complexes. Scar bar = 50 μm. Arrowheads demonstrate the colocalization of indicated cell specific markers with p-mTOR S2448 or S2481

The immunoreactivity of CD11b (+) monocytes and F4/80 (+) macrophages with mTORC1 and mTORC2 in the cryosections of CNV mice sacrificed on day 7 were examined by immunostaining for p-mTOR S2448 and p-mTOR S2481, respectively. Confocal imaging revealed greater co-immunoreactivity of CD11b (+) and F4/80 (+) cells with p-mTOR S2448 compared to (Fig. 4a, b). The co-localization of the CD31 (+) ECs, α-SMA (+) pericytes and cytokeratin (+) RPE cells with active mTORC1 and/or mTORC2 were also examined by immunostaining for p-mTOR S2448 or p-mTOR S2481, respectively. p-mTOR S2481 (+) cells were found to have a higher immunoreactivity with CD31, α-SMA, and cytokeratin compared with that of p-mTOR S2448 (+) cells (Fig. 4c-e).

### Effect of mTOR inhibition by rapamycin on laser-induced CNV

**Fig. 5:**
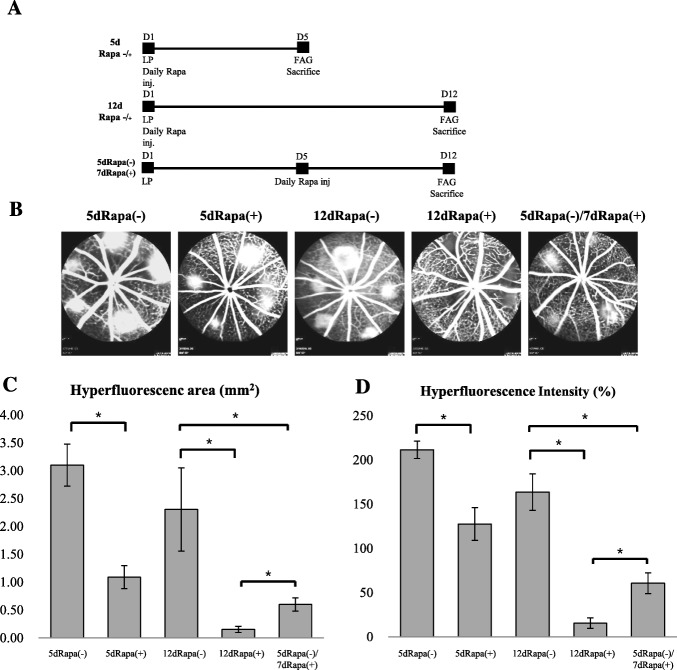
Effect of mTOR inhibition by rapamycin on laser-induced CNV. **a** – Design of Experiment B. **b** – Representative fluorescein angiograms of mice treated with rapamycin or PBS. **c**, **d** – Quantitative analysis of FFA images for hyperfluorescence area and intensity values. * - *p*-value ≤0.001. Data are presented as the mean ± S.E.M

In experiment B, we compared the preventive and therapeutic effect of rapamycin on laser-induced CNV. The prevention arm included groups of mice treated/or untreated with rapamycin during 5 days after laser exposure. Therapeutic effect of rapamycin was evaluated by comparison of CNV lesions of mice of 12dRapa(−), 12dRapa(+) and 5dRapa(−)/7dRapa(+) groups (Fig. 5a). The follow-up time was chosen based on the results of experiment A, in which the activation of the mTOR pathway occurred in parallel with the establishment of CNV on day 5 of the laser treatment. All three groups of mice in experiment B that received intraperitoneally rapamycin did not manifest any systemic side effects. Analysis of FFA images showed significantly lower values of the hyperfluorescent area and intensity in all rapamycin-treated groups (Fig. 5b-d). However, CNV lesions of mice in 12dRapa(+) group had the lowest values of hyperfluorescent area and intensity (*P* ≤ 0.001).

**Fig. 6:**
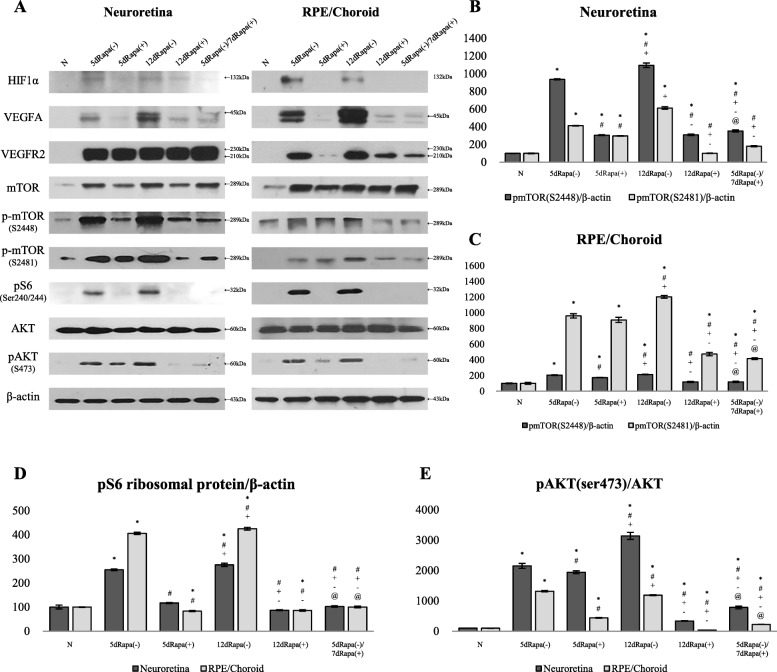
The effect of rapamycin administration on the expression of HIF1α, VEGFA, VEGFR1/2, and mTOR-related proteins detected by Western blot. **a** – Expression of indicated proteins. **b**, **c** – Comparison of expression of p-mTOR S2448 and S2481 relative to total mTOR levels in NR and RPE/Choroid. Data are presented as the mean ± S.E.M. **d**, **e** – relative expression of the pS6 ribosomal protein and pAKT (S473) normalized to β-actin. Data are presented as the mean ± S.E.M. * *P* < 0.05, Mann-Whitney U test, compared with Normal group. # *P* < 0.05, Mann Whitney U test, compared with the 5dRapa(−) group. + *P* < 0.05, Mann Whitney U test, compared with 5dRapa(+) group. - *P* < 0.05, Mann Whitney U test, compared with the 12dRapa(−) group. @ *p* < 0.05, Mann Whitney U test, compare with 12dRapa(+) group

Further, we analyzed the expression of HIF1α, VEGFA, and VEGFR2 using WB (Fig. 6a). Rapamycin treatment prevented the upregulation of HIF1α and VEGFA levels in both NR and RPE/Ch. Expression of VEGFR2 was downregulated by rapamycin treatment only in RPE/Ch.

WB analysis of samples of NR demonstrated that rapamycin significantly decreases the levels of p-mTOR S2448 and p-mTOR S2481, as well as total mTOR in both 5d and 12d groups (Fig. 6a and b). In the RPE/Ch, p-mTOR S2448 levels were significantly lower in all rapamycin-treated groups compared with that of untreated controls, of note, the results of the treatment arm demonstrated significantly lower values (*P* ≤ 0.05). mTORC2 activity was also modified by rapamycin administration in the treatment arm, p-mTOR S2481 levels were significantly decreased in both 12dRapa(+) and 5dRapa(−)/7dRapa(+) groups (P ≤ 0.05; Fig. 6c).

The expression of a downstream effector of mTORC1 – pS6 was significantly decreased in all rapamycin-treated groups in both NR and RPE/Ch (*P* ≤ 0.05; Fig. 6d). Rapamycin administration also significantly downregulated the expression of mTORC2 substrate – p-AKT S473 in both NR and RPE/Ch (*P* ≤ 0.05; Fig. 6e).

### mTOR inhibition by rapamycin enhances the activation of autophagy in laser-induced CNV

**Fig. 7:**
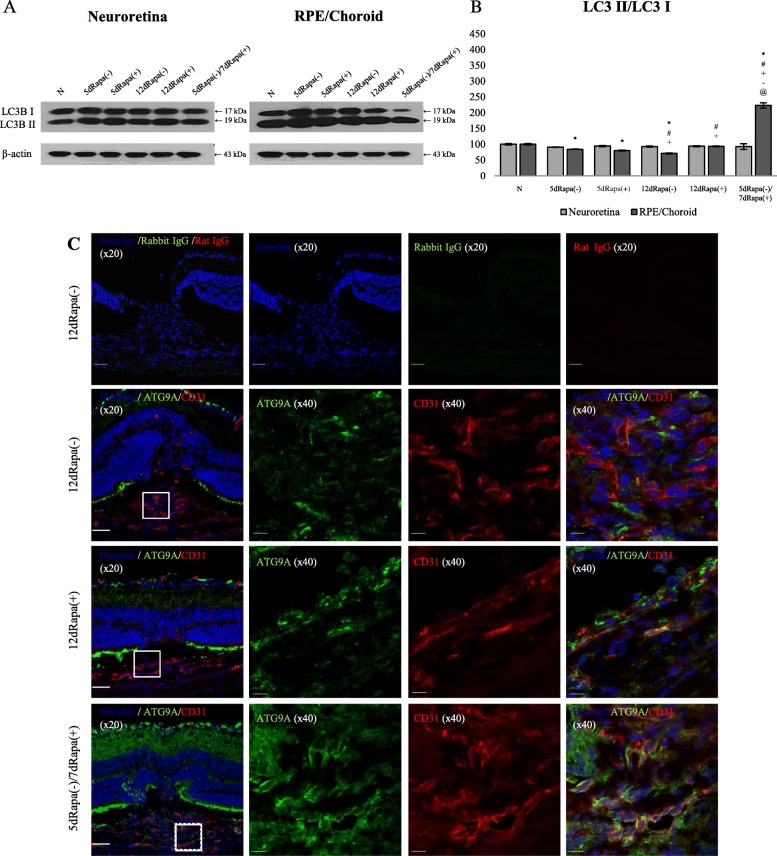
Rapamycin administration induces autophagy in the 5dRapa(−)/7dRapa(+) group. **a**, **b** - A representative Western blot analysis of LC3 and relative LC3II/I ratio normalized to β-actin. Data are presented as the mean ± S.E.M. **c** - Representative confocal images of cross sections obtained on day 12 of laser photocoagulation co-stained for ATG9A and CD31. Arrowheads indicate colocalization of ATG9A and CD31. Scale bar (× 20) = 50 μm; Scale bar (× 40) = 10 μm

To evaluate whether the autophagy activation contributes to the suppression of CNV development, we detected the level of autophagy-specific microtubule-associated protein light chain 3 (LC3) using WB. The analysis of LC3II/LC3I ratio demonstrated significantly higher levels of LC3II only in the 5dRapa(−)/7dRapa(+) group in the RPE/Ch, indicating the accumulation of autophagosomes (*P* ≤ 0.05, Fig. 7a and b).

Since LC3 level was modified only in the 5dRapa(−)/7dRapa(+) group, we immunostained the CNV lesions of the mice from treatment arm with ATG9A to evaluate the autophagy activity and CD31 to visualize endothelial cells. As shown in Fig. 7c, ATG9A immunoreactivity was high in samples from the 5dRapa(−)/7dRapa(+) group.

## Discussion

Choroidal neovascularization, a hallmark of advanced age-related macular degeneration, is the leading cause of irreversible blindness in Europe [22]. Investigations performed in human and in rodents have uncovered some aspects of CNV formation. CNV pathogenesis includes inflammation, angiogenesis, matrix deposition and remodeling [23, 24]. mTOR inhibition was found to be effective in the treatment of uveitis – intraocular inflammatory condition – uveitis [25], as well as in experimental models of various eye diseases with neovascular component, suggesting that mTOR is a prospective target for the treatment of CNV [20, 26, 27]. With the current study, we demonstrated for the first time the role of the mTORC1 and mTORC2 in subsequent processes after CNV initiation regarding inflammation and angiogenesis.

The laser-induced CNV mouse model is a widely used platform to study the underlying mechanisms of CNV development. This model is characterized by the laser-induced rupture of Bruch’s membrane to induce the invasion of new vessels from the choroid into the sub-retinal space [21]. The major initiator of neovascular processes in this model is thought to be inflammatory cells and partly pro-angiogenic factors [28]. Always in mind that stages may overlap, the wound-healing response in laser-induced CNV may be schematically divided into the acute, subacute and involutional stages. During the acute stage, the migration of inflammatory cells and bone-marrow derived cells to the lesion area, as well as a release of pro-angiogenic factors promote new vessels formation [29]. According to the morphologic examinations of laser-induced CNV, the peak of exudation and size of hyperfluorescent area occur between the 5th and 8th day of the laser treatment [30, 31]. Further, the stabilization and maturation of new vessels, including endothelium differentiation and pericyte recruitment occur up to 14–15 days of laser treatment, characterizing the subacute stage [23, 28, 31]. After 14–21 days, with the predominance of anti-angiogenic and anti-proteolytic agents, the involutional stage begins [23]. Experiment A was designed to investigate the role of mTOR pathway in the kinetics of wound healing response in a mouse model of laser-induced CNV. During the 12 days of follow-up, we observed acute and subacute stages of CNV development. FFA images acquired on day 5, confirmed the establishment of CNV by well-delineated hyperfluorescent areas. Consistent with previous studies [30], hyperfluorescence area and hyperpermeability values decreased on day 7 and kept at about similar levels up to day 12, however only the decrease of hyperpermeability values was statistically significant (*P* ≤ 0.05, Fig. 1).

Hypoxia, a potent angiogenic trigger in CNV development [32], stimulates hypoxia-inducible factor-1 (HIF1α) expression [33]. HIF1α is a major transcription factor responsible for the induction of key pro-angiogenic factor – vascular endothelial growth factor A (VEGF-A) and its receptors VEGFR-1, VEGFR-2 [32, 34, 35]. HIF1α expression continues until the balance between oxygen demand and supply is reached [36, 37]. In our experiments, HIF1α and VEGF-A expression was upregulated on day 3 and peaked on day 5 followed by a decrease on day 7 and 12, mirroring other studies that HIF1α/VEGF-A/VEGFR-2 pathway promotes the angiogenesis in CNV [31, 35].

The role of the mTOR pathway in the ocular pathological neovascularization is mostly studied regarding mTORC1 complex [16–18, 26, 27, 38, 39], with little attention paid to mTORC2. In our study, we investigated p-mTOR S2448 and p-mTOR S2481 levels, predominantly contained in mTORC1 and mTORC2, respectively [40]. WB analysis showed activation of both complexes as well as their downstream effectors in NR and RPE/Ch from day 5 to 12 of laser treatment. However, we observed two peaks in the expression of ribosomal pS6 in the RPE/Ch at day 3 and 12. If the latter was associated with mTORC1 expression, the former could be explained by the ERK pathway, which co-regulates the ribosomal pS6 [41]. We assessed total ERK and p-ERK levels by WB analysis, which demonstrated significant upregulation of p-ERK at day 3, supporting the hypothesis that the 1st peak of pS6 related with ERK pathway (*P* ≤ 0.05, Additional file 1: Figure S1A and B).

Histopathologic examination of CNV indicates the involvement of inflammatory cells in CNV formation [23]. Aside from immune response, circulating inflammatory cells (monocytes and macrophages) in CNV lesion, participate in angiogenesis by inducing the RPE-secreted VEGF, monocyte colonizations protein, and tumor necrosis factor-α, indicating their crucial role in CNV development and the peak of migration occurs on day 7 of laser injury [23, 42, 43]. In line with these findings, our immunofluorescence images of cryosections of CNV mice sacrificed on day 7 demonstrated the greater co-immunoreactivity of p-mTOR S2448 with inflammatory cell markers. Moreover, the fact that the peak of p-mTOR S2448 expression in WB analysis occurs at the same time point supports the hypothesis that mTORC1 mediates the inflammatory-driven angiogenesis in CNV (Figs. 2a and 4a). The process of maturation of new vessels is characterized by the recruitment of pericytes driven by ECs-secreted factors [23]. In the current study, ECs and pericytes in the CNV lesions had a greater immunoreactivity with p-mTOR S2481 indicating that the mTORC2 complex may play an important role in the maturation of new vessels in the CNV lesion. (Fig. 4b).

In Experiment B, rapamycin treatment downregulated the expression of p-mTOR S2448 and its downstream effector - pS6 in both prevention arm and treatment arm. One of the most important effects of the mTOR pathway in the promotion of pathological angiogenesis is the translation of hypoxia-inducible factors [44]. The crosstalk between the mTOR pathway and HIF1α can be briefly summarized in the following circle: mTORC1 / p70S6kinase / 4-EBP1 / HIF1α / VEGF-A / VEGFR2 / PI3K / mTORC1 [33]. WB analysis of samples of groups of Experiment B confirmed the role of mTOR signaling in the regulation of expression of HIF1α, VEGF and VEGFR2, since, the expression levels of these proteins were downregulated in all rapamycin-treated groups. Of note, rapamycin can interfere with HIF1α activation under hypoxic conditions and increases its degradation [45].

As a well-known fact, the long-term exposure to rapamycin modifies the mTORC2 levels. In the RPE/Ch, where the CNV originates, p-mTOR S2481 level was inhibited in the treatment arm. Interestingly, we observed the decrease of its substrate – pAKT S473 expression by rapamycin administration in prevention arm as well as in the treatment arm. One possible explanation to this may be that short-term treatment with rapamycin triggers dephosphorylation of rictor and sin1 but does not affect mTORC2 asS.E.M.bly [46].

There are clear evidences showing the relationship between autophagy dysfunction in the RPE cells and AMD pathogenesis [47–51], and activation of autophagy via mTOR inhibition by rapamycin treatment prevents the RPE damage from detrimental AMD-leading conditions [52–54]. In our experiments, the LC3II/LC3I ratio, a widely used indicator of autophagic flux, was significantly upregulated by rapamycin treatment in 5d + Rapa7d group. Other groups did not demonstrate any significant changes in autophagy activity. These were also confirmed by immunofluorescence staining, which showed the presence of ATG9A (+) cells only in the CNV lesions of mice from the 5dRapa(−)/7dRapa(+) group. Rapamycin-induced autophagy in endothelial cells inside the CNV lesion should be considered as therapeutic strategy to overcome the limitations of current management of AMD.

## Conclusion

Our study suggests that the mTOR pathway is a critical player during the subacute stage of CNV development through differentially acting with the mTORC1 and mTORC2 axes. Rapamycin administration may inhibit the angiogenesis in the laser-induced CNV by several pathways: through inhibition of inflammatory-driven angiogenesis by downregulation of mTORC1, through inhibition of the mTORC2 mediated activity of vascular components of CNV, and through induction of autophagy. Therefore, the treatment strategy downregulating pro-angiogenic factors and inflammatory activity along with enhancement of the autophagy by inhibition of the mTOR signaling has potential advantages over the anti-VEGF monotherapy, which is addressed only to inhibit vascular permeability and new vessel formation.

## Additional files

Additional file 1: **Figure S1.** The expression of total ERK and p-ERK detected by Western blot. A – Expression of indicated proteins. B – The expression of p-ERK (Thr202/Tyr204) relative to total ERK levels in RPE/Choroid. Data are presented as the mean ± S.E.M. (PPTX 470 kb)

Additional file 2: **Table S1.** Details on antibodies (PPTX 84 kb)

## Data Availability

The datasets used and/or analyzed during the current study are available from the corresponding author on reasonable request.

## Abbreviations

CNV: Choroidal neovascularization
ECs: Endothelial cells
FFA: Fundus Fluorescent Angiography
mTOR: the mechanistic target of rapamycin
NR/RPE/Ch: Neuroretina/RPE/choroid complex
RPE: Retinal pigment epithelium
VEGF: Vascular endothelial growth factor
WB: Western blot
