# Supplementary material for: mTORC1 and mTORC2 are differentially engaged in the development of laser-induced CNV
Source: Cell Commun Signal. 2019 Jun 14;17:64. doi: 10.1186/s12964-019-0380-0 (PMC6570852; doi:10.1186/s12964-019-0380-0)
Supplement: Supplementary file 1 — Figure S1. The expression of total ERK and p-ERK detected by Western blot. A – Expression of indicated proteins. B – The expression of p-ERK (Thr202/Tyr204) relative to total ERK levels in RPE/Choroid. Data are presented as the mean ± S.E.M. (PPTX 470 kb) [file 12964_2019_380_MOESM1_ESM.pptx]

## Slide 1
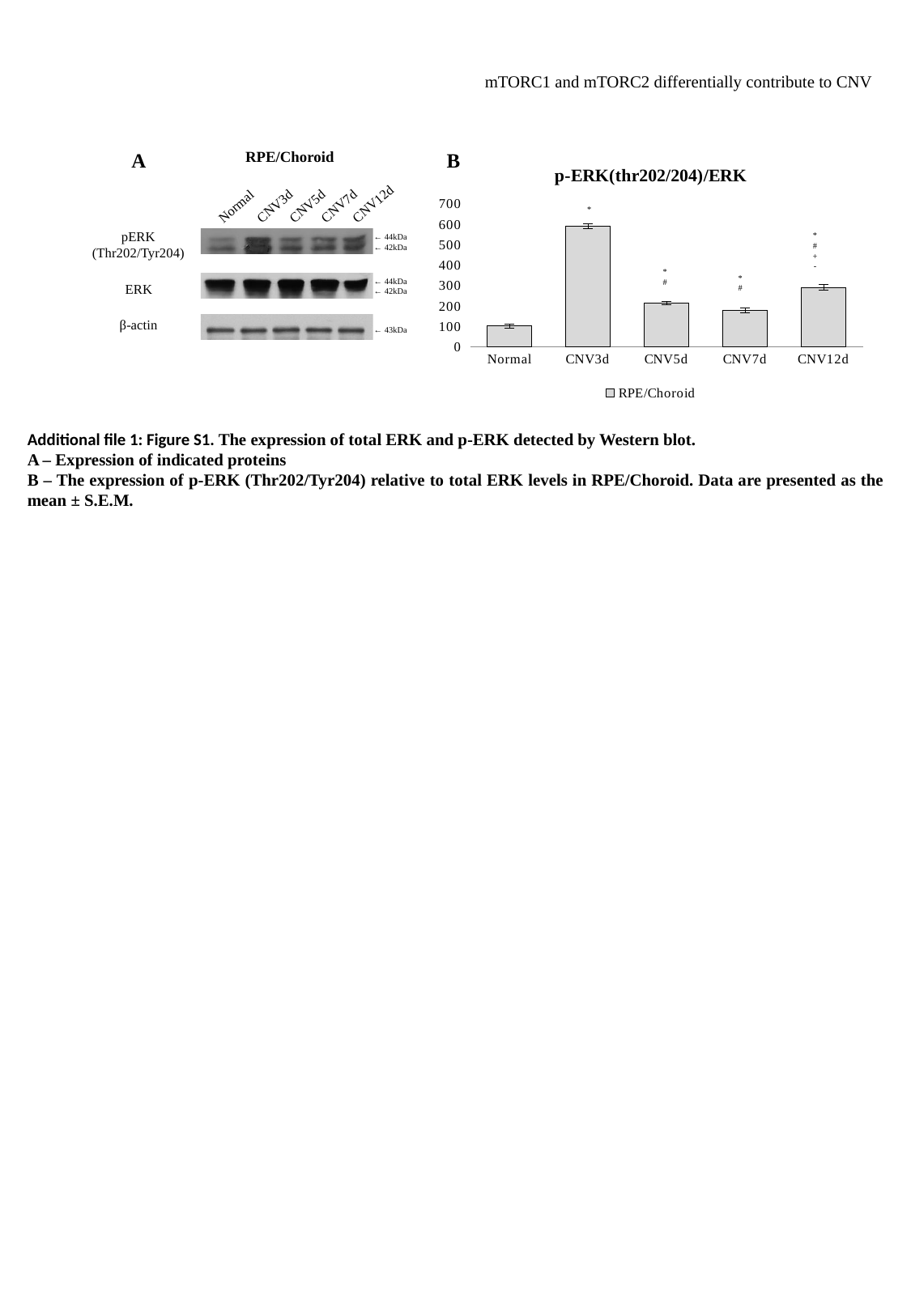

mTORC1 and mTORC2 differentially contribute to CNV
RPE/Choroid
A
B
### Chart: p-ERK(thr202/204)/ERK
| Category | RPE/Choroid |
|---|---|
| Normal | 100.0 |
| CNV3d | 590.4243749041003 |
| CNV5d | 212.11099764808753 |
| CNV7d | 178.48760707471402 |
| CNV12d | 290.0665444531167 |Normal
CNV3d
CNV5d
CNV7d
CNV12d
*
pERK
(Thr202/Tyr204)
*
#
+
-
← 44kDa
← 42kDa
*
#
*
#
← 44kDa
← 42kDa
ERK
β-actin
← 43kDa
Additional file 1: Figure S1. The expression of total ERK and p-ERK detected by Western blot.
A – Expression of indicated proteins
B – The expression of p-ERK (Thr202/Tyr204) relative to total ERK levels in RPE/Choroid. Data are presented as the mean ± S.E.M.
