# Supplementary material for: mTORC1 and mTORC2 are differentially engaged in the development of laser-induced CNV
Source: Cell Commun Signal. 2019 Jun 14;17:64. doi: 10.1186/s12964-019-0380-0 (PMC6570852; doi:10.1186/s12964-019-0380-0)
Supplement: Supplementary file 2 — Table S1. Details on antibodies (PPTX 84 kb) [file 12964_2019_380_MOESM2_ESM.pptx]

## Slide 1
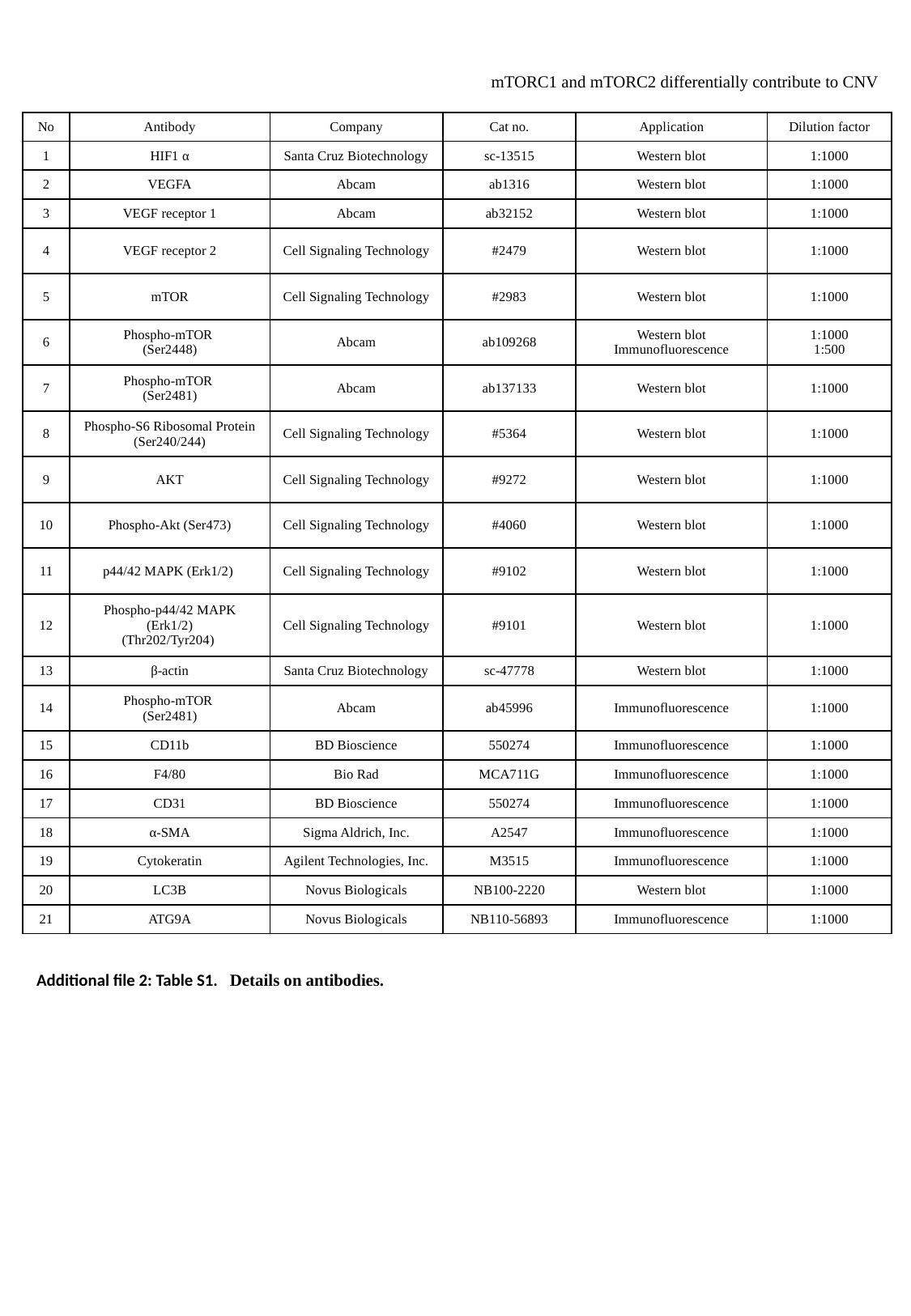

mTORC1 and mTORC2 differentially contribute to CNV
| No | Antibody | Company | Cat no. | Application | Dilution factor |
| --- | --- | --- | --- | --- | --- |
| 1 | HIF1 α | Santa Cruz Biotechnology | sc-13515 | Western blot | 1:1000 |
| 2 | VEGFA | Abcam | ab1316 | Western blot | 1:1000 |
| 3 | VEGF receptor 1 | Abcam | ab32152 | Western blot | 1:1000 |
| 4 | VEGF receptor 2 | Cell Signaling Technology | #2479 | Western blot | 1:1000 |
| 5 | mTOR | Cell Signaling Technology | #2983 | Western blot | 1:1000 |
| 6 | Phospho-mTOR (Ser2448) | Abcam | ab109268 | Western blot Immunofluorescence | 1:1000 1:500 |
| 7 | Phospho-mTOR (Ser2481) | Abcam | ab137133 | Western blot | 1:1000 |
| 8 | Phospho-S6 Ribosomal Protein (Ser240/244) | Cell Signaling Technology | #5364 | Western blot | 1:1000 |
| 9 | AKT | Cell Signaling Technology | #9272 | Western blot | 1:1000 |
| 10 | Phospho-Akt (Ser473) | Cell Signaling Technology | #4060 | Western blot | 1:1000 |
| 11 | p44/42 MAPK (Erk1/2) | Cell Signaling Technology | #9102 | Western blot | 1:1000 |
| 12 | Phospho-p44/42 MAPK (Erk1/2) (Thr202/Tyr204) | Cell Signaling Technology | #9101 | Western blot | 1:1000 |
| 13 | β-actin | Santa Cruz Biotechnology | sc-47778 | Western blot | 1:1000 |
| 14 | Phospho-mTOR (Ser2481) | Abcam | ab45996 | Immunofluorescence | 1:1000 |
| 15 | CD11b | BD Bioscience | 550274 | Immunofluorescence | 1:1000 |
| 16 | F4/80 | Bio Rad | MCA711G | Immunofluorescence | 1:1000 |
| 17 | CD31 | BD Bioscience | 550274 | Immunofluorescence | 1:1000 |
| 18 | α-SMA | Sigma Aldrich, Inc. | A2547 | Immunofluorescence | 1:1000 |
| 19 | Cytokeratin | Agilent Technologies, Inc. | M3515 | Immunofluorescence | 1:1000 |
| 20 | LC3B | Novus Biologicals | NB100-2220 | Western blot | 1:1000 |
| 21 | ATG9A | Novus Biologicals | NB110-56893 | Immunofluorescence | 1:1000 |
Additional file 2: Table S1. Details on antibodies.
